# Cognitive-Behavioral Coping, Illness Perception, and Family Adaptability in Oncological Patients with a Family History of Cancer

**DOI:** 10.1155/2017/8104397

**Published:** 2017-03-23

**Authors:** Roxana Postolica, Magdalena Iorga, Florin Dumitru Petrariu, Doina Azoicai

**Affiliations:** ^1^Department of Oncogenetics, Faculty of Medicine, University of Medicine and Pharmacy “Grigore T. Popa” of Iasi and Institute of Oncology, Iasi, Romania; ^2^Behavioral Sciences Department, Faculty of Medicine, University of Medicine and Pharmacy “Grigore T. Popa” of Iasi, Iasi, Romania; ^3^Department of Preventive Medicine and Interdisciplinarity, Faculty of Medicine, University of Medicine and Pharmacy “Grigore T. Popa” of Iasi, Iasi, Romania; ^4^Department of Oncogenetics, Faculty of Medicine, University of Medicine and Pharmacy “Grigore T. Popa” of Iasi, Iasi, Romania

## Abstract

*Aim*. The study investigated the differences between patients with and without a family history of cancer regarding coping strategies, illness perception, and family adaptability to the disease.* Material and Methods*. A total of 124 patients diagnosed with cancer were included in the research (55 of them with a family history of cancer). The* Cognitive Emotion Regulation Questionnaire*, the* Strategic Approach to Coping Scale*, the* Family Adaptability and Cohesion Scale*, and the* Illness Perception Questionnaire* were applied. The data were processed using the SPSS 21 software.* Results*. Patients with previous records of cancer in the family get significantly higher scores for the illness coherence factor. Family satisfaction is significantly higher for patients with a genetic risk, compared to the one reported by patients who suffer from the disease but have no genetic risk. Cognitive-behavioral coping strategies and family cohesion are factors that correlate with an adaptive perception of the illness in the case of patients with a family history of cancer.* Conclusion*. Results are important for the construction of strategies used for patients with a family history of cancer.

## 1. Introduction

Living under the influence of stress factors that are associated with being diagnosed with cancer demands the development of coping strategies that help the patient regain a* sense of balance* [1]. Adapting to cancer is similar to adapting to a major stress event and can be analyzed from the point of view of the intensity of the strain on the adaptive resources of the individual [2] and on the coping resources or on self-efficacy [3].

Adapting to the oncological disease depends on a series of factors, such as the individual's illness representations, the psychoemotional implications of the disease, the specific clinical characteristics of the disease and its evolution, the presence of a history of direct contact with the disease through family members, and the sociocultural context [4].

Most studies which set out to investigate the relationship between the perception of the illness and the coping strategies used by oncology patients used instruments based on the division of coping strategies into problem-focused coping (active-passive) and emotion-focused coping (positive-negative). Leventhal's Self-Regulatory Model (SRM) is the cognitive-affective model which emphasizes the existence of the emotional and cognitive components, which play a role in the perception of the threat of disease and which influence each other. Leventhal et al. [5] consider that the representation of health threats, at an individual level, is updated and enriched by actions aimed at health promotion, risk perception, and disease prevention, by disease management behaviors. The model explains that problem-focused coping focuses on resolving the stressful situation or altering the source of stress, taking control of stress by solving the problem or eliminating the source of stress, seeking information or assistance in handling the situation, and removing oneself from the disturbing situation. Emotion-focused coping is focused on managing the emotions associated with the situation, rather than changing it [6]. But drawing this distinction generates a major conceptual problem, as there is a dimension that goes beyond the limits of this dichotomy: the cognitive dimension (thoughts) versus the behavioral one (behaviors) [7, 8], which is less explored in the other studies of coping strategies for oncology patients, especially for those with a family history of cancer.

In this study, performed on oncological patients and healthy individuals with a family history of cancer, we used the model combining problem-focused active-passive behavioral coping, addressed directly to the stressor, and described in the following dimensions: active-passive, prosocial-antisocial, direct-indirect (giving the person the possibility of directly or actively confronting the causes of the stressful situation or of avoiding them, to retreat from this situation), with cognitive coping strategies centered on emotions (giving the possibility of cognitive adjustment of emotional responses to events with aggravating consequences for individual emotions).

Previous literature in the USA shows that the cognitive coping strategy contributes to the management and regulation of emotions aimed at adapting to the illness, in order to avoid patients' becoming overwhelmed by the negative event they are facing, such as the oncological diagnosis in the context of a family history of cancer [9].

On the other hand, as asserted by Edwards and Clarke in their study about levels of depression and anxiety in newly diagnosed adult patients and their adult relatives [10], cancer affects not just the patient but family functioning, family members' communication, roles and interactions, clinical levels of depression, and severe levels of anxiety and stress reactions being ascertained; therefore research and health service providers should focus on the family not just on the individual. Another study about long-term effects of cancer treatment, performed on the wives of oncological patients, reported a similar level of distress and negative consequences of the diagnosis and intervention on intrafamilial interactions, changes in family roles and communication difficulties, just as in families without a history of cancer [11], families with a history of cancer who were able to act openly, to express feelings directly, to manage problems effectively, and to communicate information directly within the family, had lower levels of depression and anxiety.

In the case of oncogenetic counseling for hereditary breast/ovarian cancer, families perceived higher anxiety and depression at genetic test result communication even if, at baseline, families were perceived by subjects as functional; in other words, they were pleased with their own family [12]. At the same time, in the case of hereditary cancer, the assessment of the family history is very important for determining risk. Thus, the family health history of cancer is considered an important instrument for prevention and health promotion. Better family cohesion and flexibility correlated with better communication between family members and better disclosure of information regarding the history of cancer [13]. In addition to a low level of information and education, poor family communication is also considered to be a barrier related to collecting data on the family history of cancer by health service providers [14].

This study aims to analyze the cognitive/emotional coping strategies of cancer patients with and without a family history of cancer and the way in which these coping mechanisms influence the perception of the illness in the context of the family system. The importance of this study comes from the fact that, to date, no study has emphasized the underlying cognitive and behavioral mechanisms used by patients with a family history of cancer in order to cope with the illness, the perception of the illness, and the characteristics of these patients' family system, in what concerns communication, family cohesion, and flexibility. Therefore, the paper outlines several aspects related to the profile of strategic coping used for adapting to illness by patients with a family history of cancer. The goal of the study is to identify the main coping strategies of patients with a genetic risk of cancer and to analyze the impact of the diagnosis on family adaptability and cohesion, by comparing the coping strategies of patients with and without a family history of cancer.

## 2. Material and Methods

The research has the approval of the Ethics Committee at the “Gr. T. Popa” University of Medicine and Pharmacy in Iasi. Between January and May 2016, 200 questionnaires were sent to oncology patient associations in 6 Romanian counties. The patient selection criteria took into account age (over 18), the unimpeded ability to understand information, the absence of cognitive, personality, or psychiatric disorders, and the completion in full of the received questionnaires. The 200 patients voluntarily took part in the research and agreed to complete our questionnaires, they were informed about the purpose of the investigation and data privacy was ensured; before filling in the questionnaire, they all signed an informed consent. A number of 124 of these questionnaires were filled in fully and returned.

As the patients come from various regions of the country, we may consider that this study analyzes a heterogeneous group of individuals from the point of view of sociodemographic data and it is not limited to the analysis of a closely defined region of the country.

After all the participants in the research provided their informed consent, sociodemographic and medical data were filled in and tests were self-administered. All these participants went through a comprehensive medical evaluation and genetic testing that provided information about their illness and their genetic risk for cancer. For a better understanding and a useful description of the data, it is important to note that the patients with a genetic risk of cancer are a subgroup of the patients with a family history of cancer.

The questionnaire included a set of specific questions designed to record sociodemographic characteristics such as sex, age, marital status, level of education, parenthood, and medical data like genetic risk, type of therapy, age at the time of diagnosis, and time since the diagnosis. In addition to this, they responded to four questionnaires assessing their coping mechanisms, illness perception, and family situation in connection with their illness,

*Cognitive Emotion Regulation Questionnaire *(CERQ [15]), the Romanian version, comprising of 36 items, is a multidimensional questionnaire that measures the cognitive coping strategies at nine different levels: self-blame, other-blame, rumination, catastrophizing, putting things into perspective, positive refocusing, positive reappraisal, acceptance, and refocusing on planning; respondents were asked to indicate to what extent they used certain cognitive coping strategies on a 5-point Likert scale (1 = (almost) never, 2 = sometimes, 3 = usually, 4 = often, and 5 = (almost) always). The sum of shares of the four items included in a scale may vary from 4 (the strategy is never used) to 20 (the cognitive coping strategy is often used); the total score of the scale has values from 4 to 20.

*The Strategic Approach to Coping Scale* (SACS), in Romanian [16], is a scale that is comprised of 52 items, that evaluates behavioral coping strategies on a 5-point Likert scale (1 = it is not at all what I would do; 5 = it is what I would do, categorically); the items are organized on nine subscales: assertive action, social joining, seeking social support, cautious action, instinctive action, avoidance, indirect action, antisocial action, and aggressive action.

*The Family Adaptability and Cohesion Scale*—FACES IV [17]—is a self-report questionnaire including 62 items organized in six subscales, that evaluates the mid ranges of adaptability and cohesion of the family (two subscales) and the extremes of adaptability and cohesion—rigid, chaotic, disengaged, and enmeshed (four subscales); respondents must express appraisals on a 5-point Likert scale, from strong disagreement (1) to strong agreement (5) and a percentile score must be used for the results (%), as well as specific computation formulas: the dimension scores for cohesion and flexibility are only used for plotting the one location of the family onto the updated graphic representation of the* Circumplex Model of Couple and Family Systems*.

*Illness Perception Questionnaire* revised (IPQ-R [18]) is a method that measures the cognitive representation of illness; it contains 38 items organized on five scales assessing* identity*, the symptoms the patient associates with the illness; the* cause*, personal ideas about etiology;* timeline*, the perceived duration of the illness; the* consequences*, the expected effects and outcome;* cure control*, how one controls or recovers from the illness; respondents were asked to choose from 1–5 answers (1 = strongly disagree, 2 = disagree, 3 = neither agree or disagree, 4 = agree, and 5 = strongly agree); it is recommended to start analysis with separate items by grouping variables; high scores on the identity, timeline, consequences, and cyclical dimensions represent strongly held beliefs about the number of symptoms attributed to the illness, the chronicity of the condition, the negative consequences of the illness, and the cyclical nature of the condition; high scores on the personal control, treatment control, and coherence dimensions represent positive beliefs about the controllability of the illness and a personal understanding of the condition.

### 2.1. Statistical Analysis

A descriptive analysis was conducted to report demographic, social, and illness characteristics of the subjects enrolled. The Pearson product-moment coefficient was used to measure the multivariate correlation between the time elapsed between the diagnosis and 4 FACES factors: disengaged, enmeshed, rigid, and cohesion ratio (2-tailed, *p* < 0.05), considering all types of patients (cured patients, patients undergoing treatment, and patients undergoing posttreatment monitoring). Linear regression analysis was used to test whether the length of treatment predicted the increase in unbalanced family relationships for the families of cancer patients. Student's* t*-test comparison was calculated for the 124 oncological patients on the criteria of the family history of cancer and coping strategies (CERQ self-blame, CERQ acceptance, CERQ rumination, CERQ positive refocusing, CERQ catastrophizing, CERQ other-blame, SACS indirect action, SACS antisocial action, CERQ positive refocusing, CERQ refocusing on planning, CERQ positive reassessment, and CERQ putting things into perspective) as dependent variable (2-tailed, *p* < 0.05). The Pearson product-moment coefficient was also used to measure the multivariate correlation between the time elapsed between diagnosis and 4 FACES factors: disengaged, enmeshed, rigid, and cohesion ratio (2-tailed, *p* < 0.05), considering all types of patients (cured patients, patients undergoing treatment, and patients undergoing posttreatment monitoring). The Pearson product-moment coefficient was also used to measure the multivariate correlation between the 124 oncological patients on the criterion of coping strategies and the influence of family records of cancer, namely, between illness coherence, the influence of family records of cancer, coping strategies, and perception of the disease. Also, Student's* t*-test comparison was calculated for the 124 oncological patients on the criterion of the influence of the genetic risk of cancer for patients with a family history of this illness.

The data were processed and analyses were performed, including all the subjects, with the SPSS package (version 21.0, IBM, USA, 2014).

## 3. Results

### 3.1. Sociodemographic and Medical Data

At the moment of data gathering, 39.52% of the 124 patients were declared cured, while 41.13% were still undergoing treatment. Only 19.35% of the participants were undergoing posttreatment monitoring. Sociodemographic and medical data are presented in Table 1.

Of all subjects, 21.8% were assessed with a genetic risk of cancer evaluated by a geneticist considering the analysis of pedigree in the context of family history of cancer. The patients were asked about their knowledge regarding genetic testing* (Table 2)*.

Almost one-third (32.45%) of them claimed that they had had no information regarding genetic testing prior to enrolling in the program, while 28.23% declared that they had had little information. Only 12.90% of participants stated they had sufficient information about genetic testing for cancer. Of all respondents, 31.5% never considered the idea that their illness could be genetically determined. Yet 59.1% of participants in the study were worried that they might carry a cancer gene that could be transmitted to their offspring. The results are presented in*Table 3*.

### 3.2. The Duration of Treatment and Its Impact on Adaptability and Family Cohesion

The time between the diagnosis and the present study correlates significantly, in patients undergoing treatment (*N* = 51), with the following FACES factors: disengaged (*r* = 0.339, *p* < 0.02), enmeshed (*r* = .524, *p* < 0.01), rigid (*r* = 0.422, *p* < 0.01), and cohesion ratio (*r* = −0.317, *p* < 0.05). The longer the duration of the illness is, the more unbalanced the family relationships become. Also, cohesion within the family tends to decrease with time in patients undergoing treatment. These correlations seem to be more significant for the group of participants still undergoing treatment (they are not statistically significant for patients who are declared cured or undergoing posttreatment monitoring). The results are presented in*Table 4*.

Linear regression analysis explores the valid model for the prediction of unbalanced family relationships for the families of cancer patients using as a predictor the variable* length of treatment*. The regression analyses were carried out for the group of patients undergoing treatment. Analyzing the relations between the predictor and criterion variables leading to the rise in unbalanced family relationships, for the sample of 51 respondents (patients undergoing treatment), namely, 1 predictor represented by the length of treatment, in turn for all 4 FACES factors (disengaged, enmeshed, rigid, and cohesion ratio), we find that each prediction model of “unbalanced family relationships” differs significantly from the previous one (*p* < 0.01).

The results obtained (Table 6) show that the variable* length of treatment* contributes significantly (*R*^2^ = 0.13; *p* = 0.015) to the explanation of the disengaged factor (*F*(1; 49) = 6.35; *p* = 0.015), contributes significantly (*R*^2^ = 0.27; *p* = 0.001) to the explanation of the enmeshed factor (*F*(1; 49) = 18.589; *p* = 0.001), contributes significantly (*R*^2^ = 0.18; *p* = 0.001) to the explanation of the rigid factor (*F*(1; 49) = 10.61; *p* = 0.001), and contributes significantly (*R*^2^ = 0.10; *p* = 0.024) to the explanation of the cohesion ratio (*F*(1; 49) = 5.461; *p* = 0.024).

From each final model, one can notice that the predictor* length of treatment* significantly influences the variance of the 4 FACES factors: disengaged (*β* = 0.339; *p* = 0.015), enmeshed (*β* = 0.524; *p* = 0.001), rigid (*β* = 0.422; *p* = 0.001), and cohesion ratio (*β* = −0.317; *p* = 0.024).

The results of the regression indicated that the length of time since diagnosis could be used as a predictor for the* FACES IV* factors: these are presented in*Table 5*.

### 3.3. Coping Strategies and the Influence of Family Records of Cancer

Comparing the results obtained by the 124 oncological patients, based on the family history of cancer, our research identified that patients with cancer scored significantly higher in the following coping strategies: CERQ self-blame, CERQ acceptance, CERQ rumination, CERQ positive refocusing, CERQ catastrophizing, CERQ other-blame, SACS indirect action, SACS antisocial action, CERQ positive refocusing, CERQ refocusing on planning, CERQ positive reassessment, and CERQ putting things into perspective.

Moreover, they scored insignificantly in the following factors: SACS assertive action, SACS social relationing, SACS seeking social support, SACS cautious action, SACS instinctive action, SACS avoidance, and SACS aggressive action.

The results obtained prove that cancer patients use acceptance and positive refocusing on planning, positive reassessment, putting things into perspective as cognitive-adaptive coping strategies. In what concerns prosocial behavioral coping strategies, cancer patients are less prone to use social relationing and seeking social support, assertive action, in addition to passive behavioral coping strategies—avoidance and indirect action. At the same time, the results reveal that, when faced with a negative event such as illness, the patients also use maladaptive cognitive coping strategies: rumination, catastrophizing, and antisocial behavioral coping strategies, such as other-blame. This could be explained by the fact that the cancer diagnosis is a factor threatening not just their health but their existence. Nonetheless, cancer patients use other-blame for the onset of the illness as a cognitive coping strategy significantly less than average for the general population* (Table 6)*.

There were significant differences between patients with a family history of cancer (M = 9.47, SD = 2.52) and patients with no such history (M = 10.66, SD = 3.18) regarding the following factors of strategic coping: indirect action [*t*(122; 121.998) = −2.27, *p* < 0.05], and there were significant differences between patients with a family history of cancer (M = 9.69, SD = 3.29) and patients with no such history (M = 11.13, SD = 4.16) regarding antisocial interaction [*t*(122; 121.998) = −2.09, *p* < 0.05]. These results support the idea that patients with a family history of cancer use antisocial action and indirect action as behavioral coping strategies less than patients with no family history of cancer. For both factors, the score mean of the genetic risk of cancer group was significantly lower.

The patients from the group with a family record of cancer (M = 6.42, SD = 2.65) scored significantly lower than those with no family record of cancer (M = 6.55, SD = 2.65) at blaming others [*t*(122; 119.620) = −1.99, *p* < 0.05] as a coping strategy. The patients from the group with a family record of cancer (M = 9.66, SD = 3.63) scored significantly lower than those with no family record of cancer (M = 10, SD = 3.55) at self-blame [*t*(122; 40.903) = −.442, *p* < 0.05] as a coping strategy. The patients from the group with a family record of cancer (M = 13.07, SD = 4.07) scored significantly lower than those with no family record of cancer (M = 13.08, SD = 4.15) at positive refocusing [*t*(122; 42.263) = −.009, *p* < 0.05] as a coping strategy.

The patients from the group with a family record of cancer (M = 9.18, SD = 4.24) scored significantly lower than those with no family record of cancer (M = 9.80, SD = 3.32) at catastrophizing [*t*(122; 35.350) = −.804, *p* < 0.05] as a coping strategy. The patients from the group with a family record of cancer (M = 14.81, SD = 2.96) scored significantly higher than those with no family record of cancer (M = 14.30, SD = 3.93) at acceptance [*t*(122; 54.243) = .620, *p* < 0.05] as a coping strategy.

The patients from the group with a family record of cancer (M = 12.81, SD = 3.11) scored significantly higher than those with no family record of cancer (M = 12.19, SD = 3.65) at rumination [*t*(122; 47.805) = .803, *p* < 0.05], as a coping strategy. The patients from the group with a family record of cancer (M = 15.37, SD = 3.15) scored significantly higher than those with no family record of cancer (M = 14.06, SD = 3.77) at refocusing on planning [*t*(122; 48.828) = 1.646, *p* < 0.05] as a coping strategy.

The patients from the group with a family record of cancer (M = 15.33, SD = 3.59) scored significantly higher than those with no family record of cancer (M = 13.33, SD = 4.23) at positive reassessment [*t*(122; 48.045) = 1.930, *p* < 0.05] as a coping strategy. The patients from the group with a family record of cancer (M = 15, SD = 3.74) scored significantly higher than those with no family record of cancer (M = 13.22, SD = 4.13) at putting things into perspective [*t*(122; 45.309) = 2.008, *p* < 0.05] as a coping strategy. There were no significant differences (*p* > 0.05) between participants with a family history of cancer and the others regarding factors of the Family Adaptability and Cohesion Scale* (FACES IV)*. When it comes to the perception of illness, patients with family records of cancer (M = 17.05, SD = 3.41) get significantly higher scores than patients with no such records (M = 15.33, SD = 4.48) for the illness coherence factor [*t*(122; 121.774) = 2.35, *p* < 0.05].

The patients from the group with a family record of cancer scored significantly lower than those with no family record of cancer at blaming others, self-blame, positive refocusing, and catastrophizing as a coping strategy, and they scored significantly higher at acceptance, rumination, refocusing on planning, positive reassessment, and putting things into perspective as coping strategies.

The data obtained emphasize that the efforts of patients with a family history of cancer to face the requirements of life as patients, assessed or perceived as exceeding or overloading their own resources, are strongly supported by coping strategies such as acceptance, rumination, refocusing on planning, positive reassessment, and putting things into perspective and less supported by blaming others, self-blame, positive refocusing, and catastrophizing, as efficient coping strategies for the situation of being ill.

Functional cognitive coping strategies ensure the regulation of emotions in oncological patients with a family history of cancer; stable ways of facing negative life events and emotional responses to situations which might worsen individual emotions are as follows: resignation to what has occurred, the continual analysis of feelings and ideas associated with the negative event, the analysis of future steps necessary to face the event, assigning positive meanings to the event, in terms of personal development, and minimizing the gravity of the event by comparison with other events.

Due to the genetic factors involved, these patients perceive the illness as being more coherent, easier to explain. They believe, to a greater extent than the group of participants with no family history of cancer, that genetic factors are the cause of their disease (*p* < 0.01, *r* = 0.52). Patients with a family history of cancer also believe more than those with no family history of cancer that an accident or a trauma caused their disease (*p* < 0.04, *r* = 0.18). On the other hand, when there is a family history of cancer, patients tend to attribute their illness to luck and chance less often than the ones with no family history of cancer (*p* < 0.03, *r* = 0.19). In what concerns the details of patients with a family history of cancer, the statistical data analysis reveals the following results:

- The adoption of a functional cognitive coping style, based on refocusing on planning (*p* = 0.0001, *r* = 0.464), positive reassessment (*p* = 0.006, *r* = 0.363), and putting things into perspective (*p* = 0.001, *r* = 0.452) correlates with an adaptive sense of illness coherence.
- Positive refocusing correlates with an adaptive emotional representation of the illness (*p* = 0.05, *r* = −0.266), in contrast to catastrophizing as a cognitive coping strategy, which correlates with a maladaptive emotional representation of the illness (*p* = 0.001, *r* = 0.431).
- Adopting a behavioral coping strategy which involves assertiveness correlates with positive control beliefs about the illness, personal (*p* = 0.015, *r* = 0.327) and through treatment (*p* = 0.005, *r* = 0.369), as well as with a sense of illness coherence (*p* = 0.012, *r* = 0.338).
- Adopting a cautious behavior is a prosocial behavioral coping strategy which correlates with a positive emotional perception of the illness (*p* = 0.007, *r* = 0.361).
- Adopting an aggressive behavior correlates weakly with control beliefs about the illness, personal (*p* = 0.031, *r* = 0.292) and through treatment (*p* = 0.006, *r* = 0.366). This result could be explained by the fact that oncological treatment, as well as the diagnosis, is perceived by patients as particularly harsh, both physically and emotionally; therefore a direct and individualistic approach when it comes to fighting the illness could be useful.
- A balanced, cohesive family system correlates with an adaptive emotional representation of the illness (*p* = 0.012, *r* = −0.338). These data are presented in*Table 7*.

*The Influence of the Genetic Risk of Cancer for Patients with a Family History of This Illness.* Patients with a genetic risk of cancer (M = 15, SD = 3.74) broaden their perspective as a coping strategy more often than patients with no genetic risk (M = 13.22, SD = 4.13), [*t*(122; 45.30) = 2.00, *p* < 0.05]. Family satisfaction seems to be significantly higher for patients with a genetic risk of cancer (M = 38.33, SD = 6.97) compared to the one reported by patients who suffer from the disease (M = 34.88, SD = 7.15) but have no genetic risk [*t*(121; 42.60) = 2.22, *p* < 0.05].

Illness coherence is significantly higher for patients with a genetic risk of cancer (M = 18.48 SD = 3.67) compared to patients with no such risk (M = 15.43 SD = 4.01), [*t*(122; 44.782) = 13.56, *p* < 0.01].

These results are confirmed by other studies in the field, showing that patients evaluated before the oncogenetic counseling are satisfied with their family adaptability, in that better family cohesion and flexibility are correlated with better communication between family members and better disclosure of information regarding family history of cancer. If problem-focused or emotion-focused coping efforts are insufficient or inadequate, patients feel fear or worry regarding disease risk; family cohesion has positive influences on the patient's coping with the disease [13]. The results are important in shaping therapeutic strategies, pointing out the importance of family involvement in the patient's recovery program* (Table 8)*.

Participants in the group with a genetic risk of cancer believe more than the other group that their illness is inherited (*p* < 0.01, *r* = 0.34). They also attribute the cause of their illness more to their own behavior than the others (*p* = 0.37, *r* = 0.19).

## 4. Discussions

The present study aims at offering a more thorough understanding of the way in which oncological patients with a family history of cancer deal with the cancer diagnosis and the way in which these patients assess coping mechanisms which influence the perception of the illness in the context of the family system: the cancer diagnosis represents a negative life event carrying a major impact on the quality of life. Adapting to the oncological disease depends on several factors: the individual's illness representations, the psychoemotional implications of the disease (characteristics and evolution), especially the presence of a history of direct contact with the disease (through family members), and the coping strategies.* Self-Regulatory Model* (SRM) is the explanatory cognitive-affective model adopted in this study, which emphasizes the emotional and cognitive components involved in disease management behaviors. The practical efficiency of these cognitive-behavioral coping strategies in accordance with SRM manifests itself in a functional perception of the disease in the context of good family communication and cohesion.

The results of this research prove that cancer patients faced illness frequently using acceptance and positive refocusing on planning, positive reassessment, putting things into perspective as cognitive-adaptive coping strategies, less often prosocial behavioral coping strategies (social relationing, seeking social support, assertive action, and passive behavioral coping strategies as avoidance and indirect action) and maladaptive cognitive coping strategies (rumination, catastrophizing, and antisocial behavioral coping strategies, such as other-blame). These data could be explained by the fact that the cancer diagnosis is a factor which threatens not just their health but their existence.

*The Self-Regulation Theory* predicted that illness representations would be directly associated with coping and, via this association, with other outcomes such as mood and disability [19]. Researchers found that a negative coping style predisposes individuals with a family history of cancer to stronger psychological distress [20]. Some studies reported that 46% of women with a family history of cancer were concerned about the possibility of developing the disease [21]. An extremely important factor is the patient's satisfaction, which is determined by a positive relationship with the doctor, good compliance, and a low rate of complications; consequently, it minimizes the psychological and physical distress.

The evidence of relationships between illness cognitions and psychological distress was proved in many researches in the field and pointed the way to the development of a psychological intervention for women diagnosed with breast cancer, based on the modification of their cognitions [22–25]. Variables such as personal history of cancer, female gender, family history of cancer, negative perception of the illness, and coping style are factors associated with maladaptive psychological manifestations (psychological distress and low level of quality of life) in people under oncogenetic surveillance for hereditary cancer [26, 27]. These variables were proved to be important factors influencing compliance with cancer screening programs. In this way, the results of this study on oncological patients promote the benefits of an early identification of individuals with a high risk of developing cancer due to family history, so that they can be included in personalized psychological intervention programs with the purpose of reducing the maladaptive coping strategies and helping them and their families deal with the diagnosis and the treatment in an adequate way. In the updated recommendations on* Genetic Cancer Risk Assessment, Counseling and Testing*, health practitioners recommend genetic counseling using: the input (medical and family histories), psychosocial assessment, cancer risk assessment (consultation and inquiring about the patient's current understanding of cancer genetic risk assessment and testing processes), genetic testing for an inherited cancer syndrome (regulations, informed consent, and counseling). The authors add follow-up considerations for better representations of the illness and coping mechanisms in order to influence the entire family system (family cohesion and family satisfaction) [28]. Other studies proved that the belief that the illness was part of family history could determine or maintain certain behaviors with negative consequences on the individual's health [29–31]. The direct connection between a high level of family cohesion and a patient's functional, adaptive representation of the disease supports the fact that, for patients with a family history of cancer, therapeutic intervention must address both patients and their families, in order to build balanced, functional systems within these families. Most people with a family history of the illness have at least some beliefs and relevant knowledge regarding their own risk of developing the illness. According to Leventhal's* Self-Regulation Model*, which supports the role of external, environmental, social, and familial factors in forming representations of the illness, these beliefs generate a cognitive-emotional and behavioral model of illness representations through which people process information and act.

The chronology, consequences, and coherence of the illness (dimensions of illness perception) have been significantly correlated with passive adaptation [32]. Simultaneously, passive adaptation has anticipated the emotional suffering caused by hereditary cancer but also concerns about cancer. In addition, significant associations have been found between the (personal and/or family) history of cancer and perceptions of cancer [33] between beliefs about inheriting cancer and adopting protective behaviors [30].

Cognitive coping, based on refocusing on planning, positive reassessment, and broadening one's perspective correlate with a high level of the sense of illness coherence, underpinning the adoption of healthy behaviors. These results thoroughly complement the results of the study conducted by Delgado in 2007, which claimed that, for chronic diseases, even when accompanied by severe physical symptoms, the positive perception of illness-generated stress and of quality of life is moderated by a strong feeling of coherence [34].

As expected, catastrophizing as a maladaptive cognitive coping strategy, frequently encountered in oncological patients, directly correlates with a dysfunctional emotional perception of the illness. At the same time, adopting prosocial behaviors for social relationing and assertive action are closely connected to positive control beliefs and an adaptive emotional perception of the illness in the case of oncological patients with a family history of cancer. These findings complete the findings of research supporting the significant correlation between coping strategies and illness perception [22, 35–38], taking into consideration the family adaptability in the context of a family history of cancer.

*Study Limitations.* The following limitations should be considered when interpreting the results of this study. Firstly, the number of women included in the research is much higher than the number of men. Secondly, this is a transversal study, whereas a longitudinal one would be more appropriate for analyzing family dynamics and the perception of illness. The third limitation is the small number of patients with a genetic risk. A higher number of patients with a family history of cancer could provide a more significant statistical analysis. The fourth limitation could be due to the self-administration of tests. Some answers could be motivated by social, cultural, and environmental representations of illness. The fifth limitation is related to the absence of scientific studies in Romania using the mentioned methodology, which makes the results have no terms of comparison and bring novelty from the perspective of psychological research to the field of oncological and oncogenetic research. This information is important for outlining worldwide intervention programs. A familiarity with the cognitive-behavioral coping strategies that correlate with a functional perception of illness enables the development of personalized psychoeducational programs for patients with a family history of cancer, in order to increase compliance with treatment, with cancer screening programs and with genetic testing. In Europe, before 2000, only United Kingdom developed such programs, but now core curriculums for genetic counseling are set up in France, Portugal, Romania, and Spain [39]. The sixth limitation is related to the small percentage of participants who declared they were still undergoing treatment or were in posttreatment monitoring, which could influence the data obtained on coping strategies and perception of the disease, considering that this perception depends on the time given by the patient to the therapeutic process. The seventh limitation is related to the possibility of assessing the level of satisfaction with quality of life of oncological patients with a family history of cancer, compared to other types of patients. This aspect will bring additional information about the impact of perception of disease, coping strategies, and the dynamics of the family system in the context of a family history of cancer.

## 5. Conclusions and Clinical Implications

Although, in Romania, there is no analysis, at the level of the individual and the family, on patients with a family history of cancer from the perspective of coping strategies and the perception of the illness in the context of the family system, the results of the research are important for clinical practice, especially when treatment and therapeutic interventions are determined. Illness perception, family adaptability, and coping mechanisms are important factors for the quality of life of a patient with a chronic disease. The success of the recovery therapy is ensured by an extended team including doctors, psychologists, and family members. Every therapeutic program must take into consideration the family history of cancer and the genetic factor, due to the fact that the representation of the disease depends on the discussed variables. The results of this study could be useful in developing screening tools to facilitate an individual and familial functioning adaptability of patients with a family history of cancer.

## Figures and Tables

**Table 1:** Sociodemographic and medical data.

Variables	Characteristics
Sex of the participants	Male: *N* = 18 (14.51%)
	Female: *N* = 106 (85.48%)

Age	M = 55.25, SD = 9.33 minimum = 27 and maximum = 70

Level of education	Primary school: 8.1%
	Secondary school: 39.5%
	High school: 14.5%
	Bachelor's degree: 37.9%

Marital status	Single (34.7%)
	Unmarried (7.3%)
	Divorced (12.9%)
	Widowed (14.5%)
	In relationship (65.3%)
	First marriage (58.95%)
	Remarried (4.8%)
	In relationship (1.6%)

The age at the time of diagnosis	M = 48.91, SD = 8.72 minimum = 27 and maximum = 70

The time since the diagnosis	1–32 years

Types of cancer	Breast cancer (65.32%)
	Colorectal cancer (11.29%)
	Ovarian cancer (6.45%)
	Cervical cancer (8.06%)
	ORL neoplasm (5.65%)
	Lung cancer (2.42%)
	Melanoma (0.81%)

Course of treatment	Surgery (94.45)
	Chemotherapy (90.3%)
	Radiotherapy (64.5%)
	Other therapy: hormonal, biological (29%)

**Table 2:** Stage of disease and family history of cancer.

Stage of disease	%	Family history of cancer	%	Genetic risk of cancer	%
Cured	39.52	Family history of cancer	44.4	With a genetic risk of cancer	21.8
Undergoing treatment	41.13	No family history of cancer	55.6	Without a genetic risk of cancer	79.2
Posttreatment monitoring	19.35				

**Table 3:** Knowledge about genetic testing and the possibility that the disease be inherited.

Knowledge about genetic testing	%	The possibility that the disease be inherited	%
No information	32.45	Worried about carrying a cancer gene	59.1
Little information	28.23	Not worried about carrying a cancer gene	41.9
Sufficient information	12.90	Never considered illness = genetically determined	31.5

**Table 4:** Correlation between FACES factors and duration of treatment.

FACES factors	Cured patients			Patients undergoing treatment			Patients undergoing posttreatment monitoring		
	*r*	*p*	*N*	*r*	*p*	*N*	*r*	*p*	*N*
Disengaged	.141	0.336	49	.339^*∗*^	0.015	51	−.178	0.406	24
Enmeshed	.098	0.503	49	.524^*∗∗*^	0.000	51	−.066	0.760	24
Rigid	.000	0.999	49	.422^*∗∗*^	0.002	51	.243	0.252	24
Cohesion ratio	−.078	0.595	49	−.317^*∗*^	0.024	51	.307	0.145	24

^*∗*^ *p* < 0.05. ^*∗∗*^*p* < 0.01.

**Table 5:** Summary of models for FACES IV factors and length of time since diagnosis.

FACES factors	*R* ^2^	df	*F*	*p*	*β*	*t*	*p*
Disengaged	0.13	1; 49	6.35	0.015	0.339	2.522	0.015
Enmeshed	0.27	1; 49	18.589	0.001	0.524	4.312	0.001
Rigid	0.18	1; 49	10.61	0.001	0.422	3.252	0.001
Cohesion ratio	0.10	1; 49	5.461	0.024	−0.317	−2.337	0.024

**Table 6:** Coping strategies and the influence of family records of cancer.

Coping strategy scale	Mean	SD	*T*	df	*p*
Self-blame	9.66	3.63	**−**.442	122	0.005
	10.01	3.55		40.903	
Acceptance	14.81	2.96	.620	122	0.036
	14.30	3.93		54.243	
Rumination	12.81	3.11	.803	122	0.024
	12.19	3.65		47.805	
Positive refocusing	13.07	4.07	**−**.009	122	0.003
	13.08	4.15		42.263	
Catastrophizing	9.18	4.24	**−**.804	122	0.023
	9.80	3.32		35.350	
Other-blame	6.55	2.65	**−**1.994	122	0.034
	6.42	2.65		119.620	
Indirect action	9.47	2.52	**−**2.270	122	0.025
	10.66	3.18		121.998	
Antisocial action	9.69	3.29	**−**2.092	122	0.038
	11.13	4.16		121.998	
Refocusing on planning	15.37	3.15	1.646	122	0.002
	14.06	3.77		48.828	
Positive reassessment	15.33	3.59	1.930	122	0.036
	13.60	4.23		48.045	
Putting things into perspective	15.00	3.74	2.008	122	0.047
	13.22	4.13		45.309	
Illness coherence	17.05	3.41	2.352	122	0.020
	15.33	4.48		121.774	

**Table 7:** Correlation between illness coherence, the influence of family records of cancer, coping strategies, and perception of the illness.

	*r*	*p*
	*The influence of family records of cancer*	
Illness coherence		
Genetic factors, cause of the disease	0.52	<0.01
Accident or a trauma caused the disease	0.18	<0.04
Luck and chance	0.19	<0.03

	*Adaptive sense of illness coherence*	
Coping strategies		
Self-blame	0.52	0.001
Positive refocusing	−0.266	0.05
Catastrophizing	−0.431	0.001
Refocusing on planning	0.464	0.001
Positive reassessment	0.363	0.006

	*Assertiveness*	
Personal control beliefs about the illness	0.327	0.015
Control beliefs about the illness through treatment	0.369	0.005
Sense of illness coherence	0.338	0.012

	*Prosocial behavioral*	
Positive emotional perception of the illness	0.361	0.007

	*Aggressive behavior*	
Personal control beliefs about the illness	0.292	0.031
Control beliefs about the illness through treatment	0.366	0.006

	*Cohesive family system*	
Adaptive emotional representation of the illness	−0.338	0.012

**Table 8:** The influence of the genetic risk of cancer for patients with a family history of this illness.

Genetic risk of cancer	Mean	SD	*t*	df	*p*
Putting things into perspective	15.00	3.74	2.00	122	0.047
	13.22	4.13		45.30	
Family satisfaction	38.33	6.97	2.22	121	0.028
	34.88	7.15		42.60	
Illness coherence	18.48	3.67	3.55	122	0.001
	15.43	4.01		44.782	
